# European *Phaseolus coccineus* L. landraces: Population Structure and Adaptation, as Revealed by cpSSRs and Phenotypic Analyses

**DOI:** 10.1371/journal.pone.0057337

**Published:** 2013-02-22

**Authors:** Monica Rodriguez, Domenico Rau, Simonetta A. Angioi, Elisa Bellucci, Elena Bitocchi, Laura Nanni, Helmut Knüpffer, Valeria Negri, Roberto Papa, Giovanna Attene

**Affiliations:** 1 Dipartimento di Agraria, Sezione di Agronomia, Coltivazioni Erbacee e Genetica, Università degli Studi di Sassari, Sassari, Italy; 2 Dipartimento di Scienze Agrarie, Alimentari e Ambientali, Università Politecnica delle Marche, Ancona, Italy; 3 Genebank Department, Leibniz Institute of Plant Genetics and Crop Plant Research, Gatersleben, Germany; 4 Dipartimento di Biologia Applicata, Università degli Studi di Perugia, Perugia, Italy; 5 Cereal Research Centre, Agricultural Research Council, Foggia, Italy; 6 Centro per la Conservazione e Valorizzazione della Biodiversità Vegetale, Università degli Studi di Sassari, Surigheddu Alghero, Italy; Instituto de Higiene e Medicina Tropical, Portugal

## Abstract

Relatively few studies have extensively analysed the genetic diversity of the runner bean through molecular markers. Here, we used six chloroplast microsatellites (cpSSRs) to investigate the cytoplasmic diversity of 331 European domesticated accessions of the scarlet runner bean (*Phaseolus coccineus* L.), including the botanical varieties *albiflorus*, *bicolor* and *coccineus*, and a sample of 49 domesticated and wild accessions from Mesoamerica. We further explored the pattern of diversity of the European landraces using 12 phenotypic traits on 262 individuals. For 158 European accessions, we studied the relationships between cpSSR polymorphisms and phenotypic traits. Additionally, to gain insights into the role of gene flow and migration, for a subset of 115 accessions, we compared and contrasted the results obtained by cpSSRs and phenotypic traits with those obtained in a previous study with 12 nuclear microsatellites (nuSSRs). Our results suggest that both demographic and selective factors have roles in the shaping of the population genetic structure of the European runner bean. In particular, we infer the existence of a moderate-to-strong cytoplasmic bottleneck that followed the expansion of the crop into Europe, and we deduce multiple domestication events for this species. We also observe an adaptive population differentiation in the phenology across a latitudinal gradient, which suggests that selection led to the diversification of the runner bean in Europe. The botanical varieties *albiflorus*, *bicolor* and *coccineus*, which are based solely on flower colour, cannot be distinguished based on these cpSSRs and nuSSRs, nor according to the 12 quantitative traits.

## Introduction

The scarlet runner bean (*Phaseolus coccineus* L., 2n  =  2x  =  22) is the third-most important *Phaseolus* species worldwide, after *P. vulgaris* and *P. lunatus* [1]. *Phaseolus coccineus* is a perennial species that can live up to 10 years, although outside Central America and Mexico it is usually cultivated as an annual, as it cannot survive frost [2], [3]. *Phaseolus coccineus* is native to Mexico, Guatemala and Honduras [4], and the wild forms are probably not all ancestral to the cultivated form. The area(s) where the domestication took place is still not known [5]. Escalante *et al.* [6] indicated that the domestication process did not erode the levels of genetic variation and that the similar levels of genetic variation among the wild and cultivated materials were mainly due to the high gene flow between the two forms. Angioi *et al.* [7] analysed a small set of *P. coccineus* accessions (wild and domesticated) using chloroplast microsatellites (cpSSRs), and they proposed two different wild genetic groups. This division paralleled the differentiation between two groups of the domesticated accessions, which suggested multiple domestication events of *P. coccineus* in Mesoamerica [7].

Based on phenotypic traits, early taxonomists described three botanical varieties of *P. coccineus:* the white-flowered type of the runner bean, known as *P. coccineus* var. *albiflorus* (DC.) Bailey; the red-flowered type, known as *P. coccineus* var. *coccineus*; and the type that has flowers of both colours (white and red), known as *P. coccineus* var. *bicolor* (Velloso) Van Eselt [8], [9]. Var. *albiflorus* has been cultivated mainly for its white seeds, while the coloured flowers of var. *coccineus* and var. *bicolor* have also led to these being grown for ornamental purposes. As a “diagnostic morphological classification” of infra-species variation [10], these three botanical varieties do not necessarily represent well-defined phylogenetic taxa.

*P. coccineus* was introduced as a crop into Europe by the Spaniards after the discovery of the Americas, probably along with the common bean, *P. vulgaris* [8]. Spain is believed to be the country of introduction of these beans into Europe, as indicated by the Italian name ‘*fagiolo di Spagna*’ (Spanish beans). After that, *P. coccineus* spread from Spain to Italy and to the other parts of the Old World [1], [11]. Its diffusion was mainly due to its ability to grow at low temperatures (down to 5 °C) [2], [3], although it is adapted to more restricted environmental conditions than *P. vulgaris*. The United Kingdom, The Netherlands, Italy and Spain appear to be the European countries where *P. coccineus* is more widespread [1]. In particular, in the United Kingdom, the runner bean has often substituted for the common bean, because it is more adapted than other *Phaseolus* species to the cold temperatures and cool summers [2], [3]. Although *P. coccineus* and *P. vulgaris* have two different mating systems (allogamous and autogamous, respectively), they are cross-fertile when *P. vulgaris* is the maternal parent [6], [12]. This incomplete reproductive isolation between *P. coccineus* and *P. vulgaris* might have allowed hybridisation between these two species in Europe, where they are often grown in sympatry [13], [14]. For this reason, the runner bean might also be a useful source of diversity for *P. vulgaris* breeding.

Relatively few studies have analysed the genetic diversity of European runner-bean landraces using molecular markers. Most of these have analysed a comparatively low number of local accessions from relatively restricted geographical areas, or have focused on the comparison of *P.vulgaris versus P. coccineus* [15]–[19], while a broader analysis was presented using nuclear microsatellites (nuSSRs) [20].

Alvarez *et al.* [21] compared Spanish and Mexican accessions of *P. vulgaris* and *P. coccineus,* and they suggested that the runner bean maintained a high level of diversity after its introduction into Europe. Other studies have suggested changes in the structure of the genetic variation, probably due to selection, to rapid adaptation to the new growing conditions, or to demographic processes, such as bottleneck and founder effects [17], [18], [20].

To gain insight into the evolutionary processes that have shaped the diversity pattern of the European runner bean, and to test for the occurrence of cytoplasmic bottlenecks following the introduction of *P. coccineus* into the Old World, we analysed an American collection and a European collection of *P. coccineus* through cpSSRs, which explore highly polymorphic sequences [7], [17]. The cpSSRs are uniparentally inherited and non-recombinant, and therefore the genotyping directly results in haplotypes determined by the combination of alleles at several cpSSR loci. Moreover, due to their haploid genome, cpSSRs are particularly efficient for the detection of bottleneck effects, and have been successfully used to analyse the phylogenetic relationships in *Phaseolus* spp., and the diversity within species [7], [17], [22], [23].

We additionally complemented these cpSSR data with a previously unpublished phenotypic characterisation that was carried out at the Leibniz Institute of Plant Genetics and Crop Plant Research (IPK) in Germany. By comparing these data, we have been able to investigate the associations between cpSSR groups and phenotypes, and also the geographical clinal variations in phenology for putative adaptive traits, such as flowering time. These results are used to infer the role that migration, drift and selection have had in the shaping of the genetic structure of the European runner bean.

For a subset of 158 individuals, we have compared and contrasted the cpSSR and phenotypic data obtained in the present study with those obtained in another study that used 12 nuSSRs [20]. This comparison has allowed the investigation of the role of gene flow, migration and recombination, based on the different inheritance modes of these two marker systems. The phenotypic traits and genetic data are finally integrated to investigate the relationships among the botanical varieties of *albiflorus*, *bicolor* and *coccineus*.

## Materials and Methods

### Plant materials

We analysed 37 American and 331 European domesticated accessions of *P. coccineus*, each of which represented a single plant (Table 1 and Table 2). Additionally, 12 wild accessions of *P. coccineus* were investigated (Table 1). The list of the accessions analysed is given in Table S1. The European landraces were from 17 different countries, although for 21 of the accessions, the origins were not known (Table 2).

**Table 1:** Distribution and type of the American accessions of *P. coccineus* used in the present study.

Country	No. of accessions		
	All	Domesticated	Wild
Costa Rica	3	3	–
Guatemala	6	1	5
Honduras	2	2	–
Mexico	38	31	7
*Total America*	*49*	*37*	*12*

**Table 2:** Distribution and type of the European accessions of *P. coccineus* used in the present study.

Country	No. of accessions			
	All	*albiflorus*	*bicolor*	*coccineus*
Albania	2	2	–	–
Austria	79	16	27	32
Bulgaria	10	8	–	2
Croatia	3	3	–	–
Georgia	23	5	–	18
Germany	14	3	–	11
Hungary	7	–	–	–
Italy	29	15	3	8
Moldova	2	2	–	–
The Netherlands	18	6	–	12
Poland	10	7	–	3
Portugal	8	–	–	–
Romania	23	8	3	7
Slovakia	35	5	14	16
Slovenia	9	–	–	–
Spain	33	–	–	–
Ukraine	5	2	2	1
No information	21	6	3	11
*Total Europe*	*331*	*88*	*52*	*121*

Our collection partially overlaps with that analysed by nuSSRs in [20], with 158 accessions shared between the two studies (43 from America, 115 from Europe). The botanical classification into the three botanical varieties of *P. coccineus, i.e.,* var. *albiflorus*, var. *bicolor*, and var. *coccineus* was available for 260 of the European accessions (with nuSSR data also available for 62 of these), and for 13 out of the 17 countries (Table 2). The five qualitative traits upon which the classification of these three varieties was based are given in Table 3 (data and botanical classification provided by IPK, Gatersleben, Germany).

**Table 3:** Phenotypic differences among the three varieties of *P. coccineus* used in the present study.

Variety	Flower colour	Seed			Stem colour
		Colour	Pattern colour	Hilum colour	
*albiflorus*	white	white	none	none	green
*bicolor*	red and white	beige	brown	none/beige	green/red
*coccineus*	red	purple	black	black	red

For these 260 European accessions, the IPK provided the geographic coordinates, when available, plus additional quantitative phenotypic traits. The seed multiplication and characterisation of the different *P. coccineus* accessions started at the IPK in 1945 to 1946, in small-plot experiments in the field or in greenhouses. These took place every two to three years until 1976, when a cold-storage facility was established. Afterwards, the average frequency of seed multiplication dropped to once every 10 years to 30 years, when either the germination rate or the amount of seed available (*e.g.*, due to seed distribution) dropped below a critical threshold. Phenotypic observations were recorded, to document the traits of the individual accessions, and to be able to compare plants of the same accession over different years. Each accession was characterised across a different number of years, with an average of data across three years. The phenotypic traits used in the present study include phenological traits, such as the sowing, emergence and flowering (onset and end) dates, and morphological traits, such as the pod length, width and depth, the number of seeds per pod, seed length, width and depth, and thousand-seed weight. From these data, we calculated the intervals of the days from sowing to flowering, from emergence to flowering, and from onset to the end of flowering (*i.e.*, the flowering interval). Seed volumes were also calculated from the seed sizes provided. A list of the mean values of all of the 12 phenotypic traits used for the present analysis is given in Table S2.

Phenotypic data were not available for samples from Hungary, Portugal, Slovenia and Spain.

The European accessions were provided by the following Institutes: IPK, Germany; Banco Português de Germoplasma Vegetal (BPGV), Portugal; Instituto de Investigaciones Agropecuarias (INIA), Chile; Consejo Superior de Investigaciones Científicas (CSIC), Spain; and Dipartimento di Biologia Applicata (DBA), University of Perugia, Italy. The American accessions were provided by the United States Department of Agriculture (USDA), USA (Table S1).

### DNA extraction and cpSSR analysis

Young leaves were harvested for DNA extraction, which was carried out on a single plant basis, using DNeasy 96 plant kits and an MM300 Mixer Mill (Qiagen GmbH, Hilden, Germany).

The European and American accessions were analysed for the six cpSSRs used in [22], [23] to study the European landraces of the common bean (ccSSR2, ccSSR9, ccSSR11, ccSSR16, ccSSR19, ccSSR20), and also using information and protocols described by Angioi [7]. The nuSSR data were provided by the University of Perugia; for these methods, see [20].

### Statistical analyses

Correlations among phenotypic traits and between traits and geographic variables (*i.e.*, longitude, latitude, altitude) were calculated as Pearson’s *r* coefficients. Differences among botanical varieties or among genetic groups (cpSSRs, nuSSRs) for the different traits were tested by one-way ANOVA. Multivariate analysis was also carried out on the phenotypic data, through principal component analysis (PCA). The principal components were derived from eigenvalue decomposition of the correlation matrix, and the principal component points were derived from the eigenvector linear combination of the standardised variables. All of these analyses were performed using Jump, version 7.0 [24].

The cpSSR gene diversity was estimated using Nei’s unbiased gene diversity [25]. Multilocus diversity was estimated using the normalised Shannon diversity index for haplotype frequencies (*I_nor_*_,_) [26]. To compare the number of alleles for samples of different sizes, we calculated the allelic richness (*R_s_*), using the rarefaction method of [27], and implemented in the FSTAT software, version 2.9.3.2 [28].

The genetic divergence between the groups of accessions (continents, regions within the European continent) was estimated using the *F_ST_* statistic [29], using FSTAT [28]. The significance of *F_ST_* was assessed by randomising the genotypes among the samples (10,000 randomisations), while not assuming random mating within samples, and using the log-likelihood G [30].

The existence of a bottleneck between America and Europe was determined by the method defined by [31] for *Zea mays*, and as already used with *P. vulgaris* [22], [23]. In more detail, we calculated the relative deficit of given statistics in Europe *versus* America (*e.g.*, number of rare alleles, number of alleles, allele richness, gene diversity, multilocus diversity). For example, to detect the deficit for gene diversity (GD) we used the parameter ΔGD  =  1 - (*H_E_/H_A_*), where *H_E_* and *H_A_* indicate the genetic diversity in Europe and America, respectively, and where *H_A_ > H_E_*_._ If *H_A_* < *H_E_*, we calculated this parameter as ΔGD  =  -[1-(*H*_A_ */H_E_*)]. These statistics vary between -1 and 1: when ΔGD is positive, the diversity is higher in America, and when ΔGD is negative, the diversity is higher in Europe.

The population structure was determined by applying a non-spatial genetic mixture analysis to the cpSSRs, using the linked loci option and assuming independent loci, as implemented in the BAPS software, version 5.1 [32], [33]. We carried out genetic mixture analysis at the individual level, by performing 20 iterations of K (from 1 to 20), to determine the most probable number of populations (K). The nuSSR data were also analysed with BAPS according to [32], to allow direct comparisons with the cpSSR data. Under its default settings, BAPS identified the best partition of the data as that with the highest marginal log-likelihood (21 groups for cpSSRs, 24 groups for nuSSRs). As also observed in previous studies, *e.g.*, [34], we observed a tendency of BAPS to overestimate the most likely number of Ks, as the majority of individuals were assigned to a small number of large clusters (Figure S1). Therefore, we also ran the data using the fixed K option (from two to six), to only retain the groups that represent the uppermost hierarchical levels of the genetic structure. The UPGMA trees constructed with the Kullback–Leibler divergence matrix provided as an output of the BAPS software are also illustrated.

The significance of the correlations between distance matrices (cpSSRs, nuSSRs, phenotypic, geographic) were calculated using the Mantel’s test [35], with GenAlEx, version 6 [36].

## Results

### Chloroplast genetic diversity in America and Europe

Overall, the cpSSR analysis identified 22 alleles across the 380 accessions, ranging from two (ccSSR2) to five (ccSSR19) (Table S3). In America, the wild gene pool diversity was higher than the domesticated gene pool diversity for allelic richness, gene diversity and haplotype diversity (Table 4).

**Table 4:** CpSSR genetic diversity summary statistics for the American and European samples used in the present study.

Accession	Sample size	Allele number		R_S_	Haplotype number		Hap/Ind	*H_E_*	*I*_nor_
		n_a_	n_a__<0.1_		Total	Unique			
America	49	19	6	–	22	19	44.90	0.42	0.70
*Domesticated*	*37*	*16*	*4*	*(16.00) [11.8]*	*15*	*8*	*40.54*	*0.33*	*0.64*
*Wild*	*12*	*17*	*3*	*[15.00]*	*10*	*4*	*83.33*	*0.56*	*0.91*
Europe	331	19	8	(14.98)	29	26	8.76	0.29	0.47
Total	380	22	13	–	48	–	12.63	0.61	0.52
*Diversity of variation between continents (Δ)* *	–	*0*	–*0.25*	*0.06*	–	–	–	*0.13*	*0.26*

n_a_ =  number of alleles, total and rare (n_a_ <0.1); R_s_  =  allelic richness; Hap/Ind  =  haplotype/accessions ratio; *H_E_*  =  Nei’s (1978) gene diversity; *I_nor_*  =  normalized Shannon-Weaver index of haplotype diversity.

Note: within the *R_s_* columns, comparisons should be made between the values enclosed between the same bracket types.

* when Δ is negative Europe > America.

Also, within the domesticated gene pool, there was a total of 22 alleles (American and European). We detected 16 alleles in the American sample, and 19 in the European sample; 13 were shared between these continents, three were unique to America, and six were unique to Europe. The allelic richness was higher in the domesticated American accessions (R_S_  =  16.00) than in the European accessions (R_S_  =  14.98), leading to a ΔR_S_ of 0.06 (Table 4). Accordingly, the gene and genotypic diversity were higher in the American domesticated accessions (*H_E_*  =  0.33; *I_nor_*  =  0.64) than in the European accessions (*H_E_*  =  0.29; *I_nor_*  =  0.47), with a Δ*H_E_* of 0.13 and a Δ*I_nor_* of 0.26 (Table 4). This provides evidence of a moderate bottleneck effect between America and Europe.

In the European accessions, there was a high percentage of unique haplotypes (26/29, 89.7%) (Table 4). The three haplotypes shared between the continents only accounted for 16.6% (55/331) of the European accessions, and 10.2% (5/49) of the American accessions. This suggests a strong population structure between these two continents.

Indeed, the genetic differentiation between the American and European continents that was detected by cpSSRs was high (*F_ST_*  =  0.369), and highly significant (P <0.0001).

### Population structure in America and Europe

**Figure 1:**
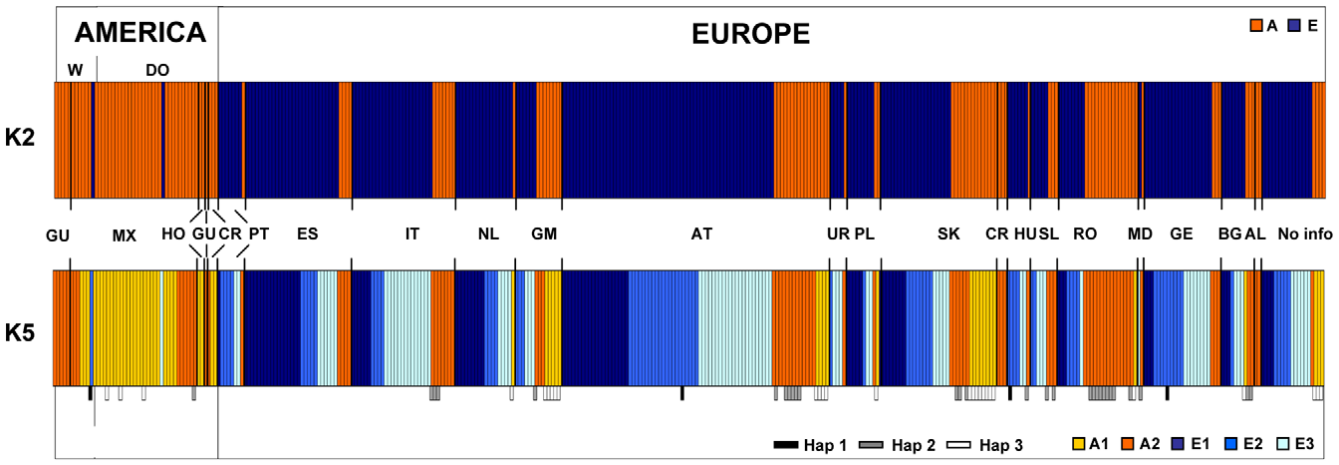
Cytoplasmic genetic structure. BAPS cluster assignments at K2 and K5 based on the cpSSR data of 331 European and 49 American accessions of *P. coccineus*. Beneath the K5 pattern, the three haplotypes shared between continents are indicated by black, grey and white bars. Abbreviations: *W* Wild; *DO* Domesticated; *GU* Guatemala; *MX* Mexico; *HO* Honduras; *CR* Costa Rica; *PT* Portugal; *ES* Spain; *IT* Italy; *NL* The Netherlands; *GM* Germany; *AT* Austria; *UR* Ukraine; *PL* Poland; *SK* Slovakia; *CR* Croatia; *HU* Hungary; *SL* Slovenia; *RO* Romania; *MD* Moldova; *GE* Georgia; *BG* Bulgaria; *AL* Albania; *No info* no information on country of origin.

Two main genetic groups were detected using BAPS (groups A and E; Figure 1). Group A includes all except two of the American accessions (47/49) and 92 of the 331 European accessions. These latter 92 accessions are present in all of the European countries. Group E includes the majority of the European accessions and only two accessions from America (one domesticated accession and one wild accession, both from Mexico).

**Figure 2:**
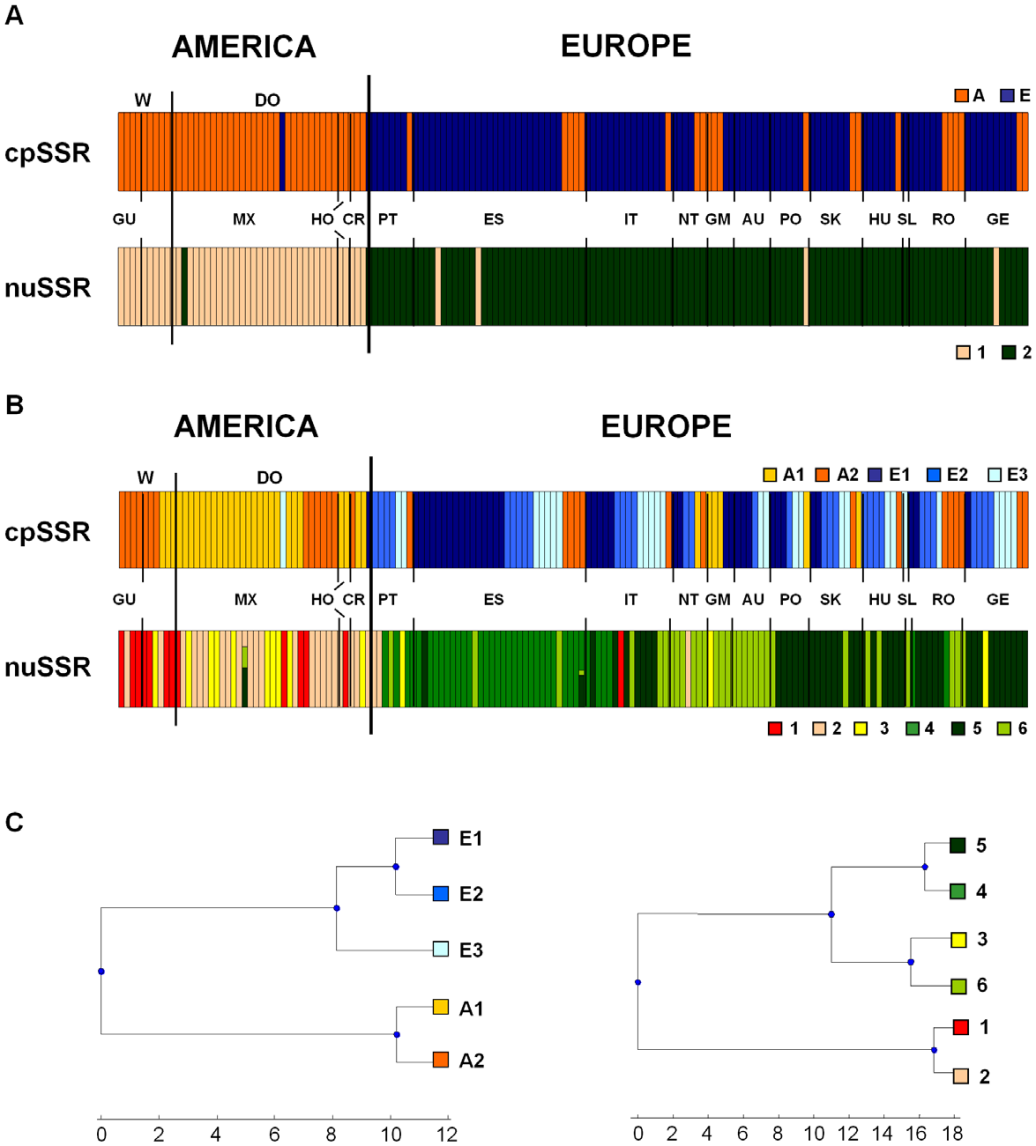
Cytoplasmic and nuclear genetic structure. Comparison between the different clusterings of the accessions as inferred by BAPS at K2 (A) and at K5 and K6 (B) for cpSSR and nuSSR data. Only accessions shared between Spataro *et al*. [20] and the present study (158) are included; (C) TFPGA tree based on cpSSR and nuSSR Kullback–Leibler divergence matrices, as obtained with BAPS analysis.

At K5, we detected groups A1, A2, E1, E2 and E3 (Figure 1). Four of these groups were from both America and Europe, although they showed very different frequencies across these continents: groups A1 and A2 were highly represented in America, and groups E2 and E3 were mostly represented in Europe. Group E1, which is close to group E2 based on genetic divergence (Figure 2C), is exclusive to Europe (Figure 1).

Group A1 mainly includes accessions from Mexico (27), as both wild (3) and domesticated (24), and four further domesticated accessions, two from Honduras and two from Costa Rica. Group A2 includes all of the accessions from Guatemala (five wild and one domesticated), nine accessions from Mexico (six domesticated and three wild), and one domesticated accession from Costa Rica. In Europe, the A1 and A2 groups are not equally represented, with 25 accessions belonging to group A1, and 67 accessions belonging to group A2.

The three haplotypes shared between America and Europe (frequencies, 7.6%, 7.1%, 1.0%) were attributed to three of the five groups (A1, A2, E2) and are present in most of the European countries, but not including Portugal, Spain, Moldova, Ukraine and Albania (Figure 1).

In Europe, groups E1, E2 and E3 have relatively high frequencies and are almost equally represented (23.6%, 23.3%, 26.0%, respectively). No association among the genetic groups and the European countries was detected. A very low but significant correlation between the cpSSRs and geographic distances was observed (r  =  0.10, P  =  0.01). Only two accessions from America were attributed to group E2 (one wild accession from Mexico) and to group E3 (one domesticated accession from Mexico).

### Distribution of genetic diversity in Europe

Across the main European areas, we observed a relatively even distribution of genetic diversity (Table 5). In the Iberian Peninsula and in Italy, we detected the highest number of alleles (16), the highest ratio of haplotypes/ accessions (26.8%), and the highest gene diversity (0.31). In central-northern Europe, we detected the lowest diversity (*H_E_*  =  0.26, *I_nor_*  =  0.53). The differentiation level (*F_ST_*) among the European countries (with >10 accessions) was low, at 0.04 (P <0.0001).

**Table 5:** Diversity across the four regions of the European continent.

Country	Sample size	n_a_	Haplotype number		Hap/Ind	*H_E_*	*I_nor_*
			Total	Unique			
Iberian peninsula + Italy	70	16	19	5	26.8	0.31	0.61
Central-northern Europe	110	13	19	1	17.0	0.26	0.53
Eastern Europe	51	12	18	0	24.7	0.28	0.66
Southeastern Europe	79	14	18	2	25.0	0.28	0.68
*Mean*	*78.25*	*13.75*	*18.5*	–	*23.3*	*0.28*	*0.62*

n_a_  =  alleles number_;_ Hap/Ind  =  haplotype/accessions ratio; *H_E_*  =  genetic diversity; *I_nor_*  =  normalised Shannon-Weaver index. Iberian Peninsula and Italy; Italy, Portugal, Spain; Central-northern Europe: Austria, Germany, The Netherlands; Eastern Europe: Georgia, Poland, Slovakia, Ukraine; South-eastern Europe: Albania, Bulgaria, Croatia, Hungary, Moldavia, Romania, Slovenia, Hungary.

### Comparisons between cpSSRs and nuSSRs in Europe

To allow direct comparisons of the cpSSRs and nuSSRs, we re-analysed the data from [20] using BAPS. Overall, there was a significant correlation between the genetic groups detected by cpSSRs and nuSSRs, both at K2 (Pearson χ^2^  =  76.48, d.f. 1; P <0.0001; Figure 2A) and at K5 (plastidial subdividion) *versus* K6 (nuclear subdivision) (Pearson χ2  =  97.78, d.f. 20; P <0.0001; Figure 2B). This was mainly due to the high genetic divergence between America and Europe (Figure 2C). Indeed, when comparing the population structure of each continent individually, we did not find any associations at either K2 or K5 (P >0.05; Table S4).

### Associations between genetic and phenotypic patterns in Europe

When looking at the phenotypic differences among the 260 accessions for which cpSSR data and phenotypic data are available, we found that at K2, the European accessions attributed to the two cpSSR groups (A and E) differ in seed size and weight, and in pod length and volume (Table 6). Interestingly, the accessions included in group A always show lower values for all of these traits.

**Table 6:** Results of the one-way ANOVA for phenotypic traits among the chloroplast groups.

Cluster assignment Group	Flowering interval (days)		Pod length (cm)		Seed length (mm)		Seed width (mm)		Seed volume (cm^3^)		Thousand-seed weight (g)	
K2												
A	73.78	a	14.12	a	20.92	a	12.67	a	2.37	a	1232.73	a
E	74.42	a	15.27	b	22.02	b	13.37	b	2.70	b	1372.74	b
K5												
A1	67.72	bc	14.82	ab	20.92	b	12.52	c	2.33	b	1250.56	cd
A2	77.95	a	13.77	b	21.02	b	12.86	bc	2.45	b	1233.06	d
E1	67.71	c	15.80	a	21.47	ab	13.26	ab	2.52	ab	1358.24	ab
E2	74.82	ab	15.60	a	22.39	a	13.07	abc	2.70	ab	1330.93	bc
E3	78.32	a	14.71	ab	22.11	a	13.58	ab	2.80	a	1410.90	a

Different letters indicate means within columns that are significantly different (P <0.05; Student t test) for each K.

This pattern is consistent with that observed at K5 (Table 6). Indeed, the accessions attributed to groups A1 and A2 have the lowest seed size and seed weight. Accessions from group A1 also have the lowest pod length. Additionally, groups A1 and A2 also differ according to their interval of flowering (67.72 days, 77.95 days, respectively), and groups E2 and E3 according to their thousand-seed weight (1330.93 g, 1410.90 g, respectively).

On the contrary, with the 62 accessions for which both nuSSRs and phenotypic data are available, the European nuclear groups (both at K2 and K6) do not show any significant differences according to the phenotypic traits (P >0.05, for all of the comparisons).

### Associations of molecular and phenological traits with geographical data

**Figure 3:**
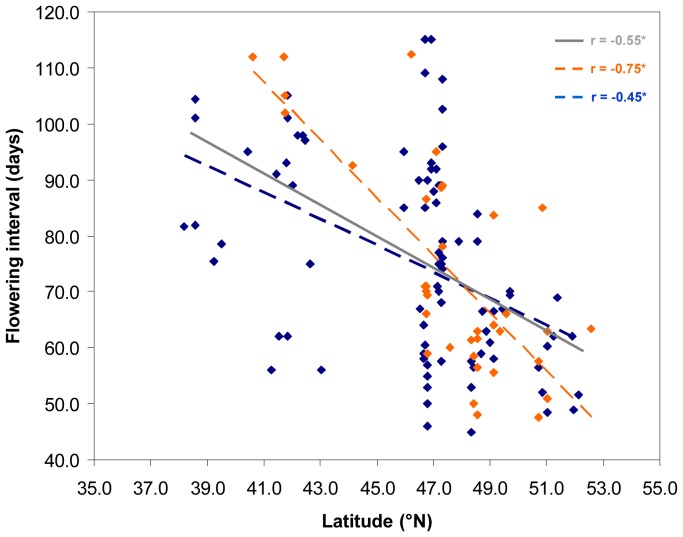
Correlation between latitude and flowering interval. Different colours indicate accessions from the two cpSSR groups A (orange) and E (blue) (see Figure 1), for which the regression lines (dashed) are illustrated. The trend line of the overall sample (grey continuous line) is also indicated. * P <0.0001

We did not find any significant associations between the days to flowering and the geography (neither for sowing, nor for emergence). On the contrary, we did find a highly significant negative correlation between latitude and interval of flowering for the overall sample (r  =  -0.55, n  = 122; P <0.0001; Figure 3) (*i.e.*, increased latitude was associated with decreased interval of flowering). Negative correlations were also found between latitude and emergence dates, and between latitude and flowering time, although these did not reach significance (P >0.05). The accessions from the chloroplast group A had a mean latitude of 48.35 °N, and those belonging to group E, had a mean latitude of 45.56 °N, and the difference in latitude between these two groups is significant (P <0.03). The association between latitude and flowering time was also confirmed when we used multiple regression to factor out the genetic structure, either as chloroplast or nuclear (Table S5). In particular, the significance of the correlation was maintained within each of the two cytoplasmic groups, A (r  =  -0.75, n  =  36, P <0.0001) and E (r  =  -0.45, n  =  86; P <0.0001) (Figure 3).

### Divergence among varieties and geographic distribution of the phenotypic traits

Among all of the 12 quantitative traits investigated, the three botanical varieties of *albiflorus*, *bicolor* and *coccineus* were significantly different only according to pod length and seed volume (Table 7). In particular, var. *bicolor* showed a greater seed volume, while var. *coccineus* showed a longer pod. However, the combination of these two traits has very low diagnostic capability. Indeed, based on linear discriminant analysis, considering these three varieties as categories and the 12 traits as covariates, only 28.6% of the accessions can be correctly assigned to their variety groups of origin.

**Table 7:** Varietal differences among the three varieties of *P. coccineus*.

Variety	Pod length (cm)	Seed volume (cm^3^)
*albiflorus*	14.58 b	2.41 b
*bicolor*	13.80 b	2.89 a
*coccineus*	15.67 a	2.60 b

Different letters indicate means within columns that are significantly different (P <0.05; Student t test).

At the molecular level, these three varieties are not significantly differentiated according to either cpSSRs or nuSSRs (*F_ST_*  =  0.007, P  =  0.45; *F_ST_*  =  0.002 P  =  0.59, respectively); moreover, the association tests performed among the varieties and the plastidial and nuclear genetic groups were not significant (Pearson χ^2^  =  11.41, n  =  8, P  =  0.18; Pearson χ^2^  =  7.61, n  = 8, P  =  0.47, respectively).

**Figure 4:**
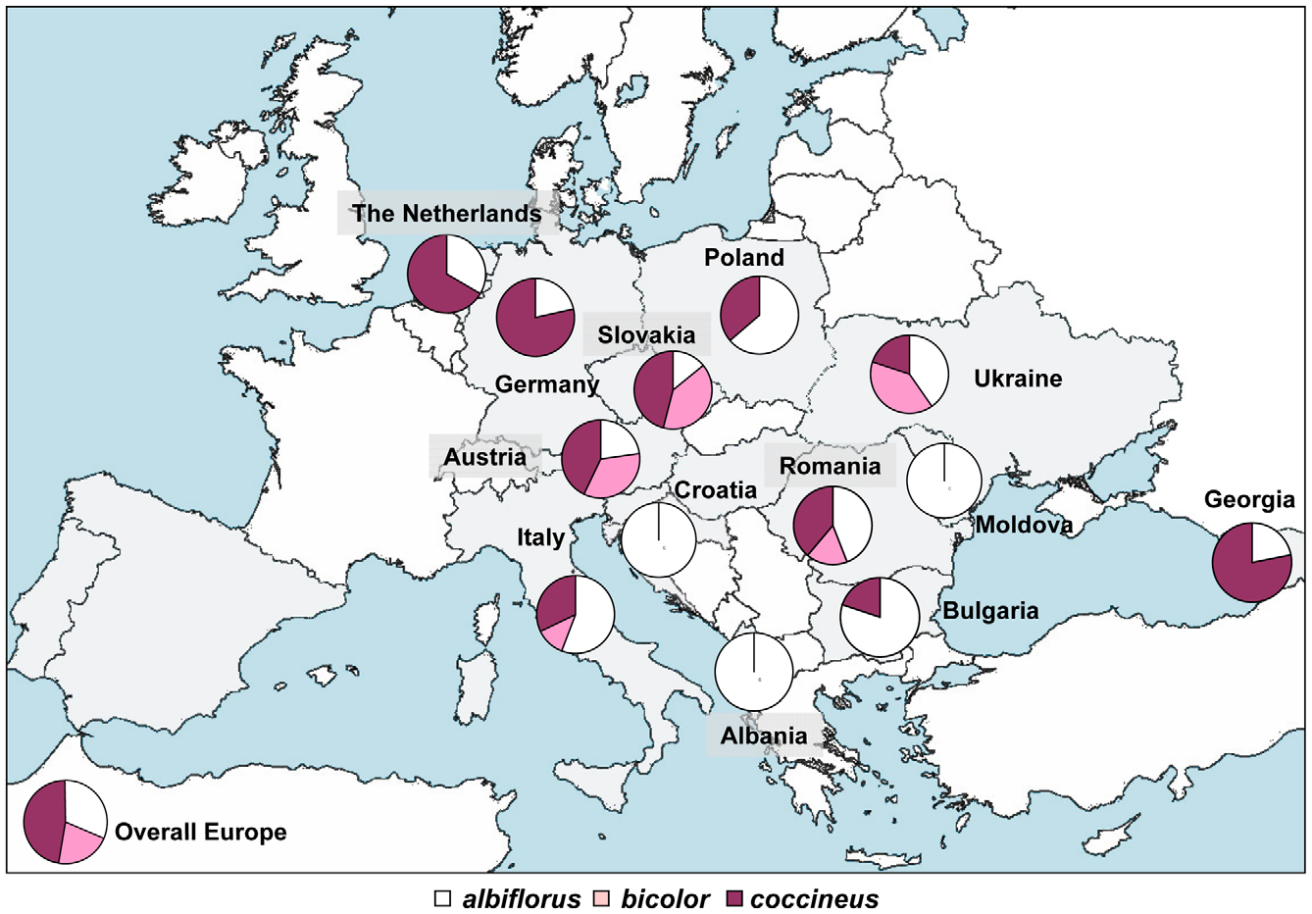
Varieties distribution across Europe. Geographic distribution of the 260 varieties identified as var. *albiflorus,* var. *coccineus* and var. *bicolor.*

The geographic distribution of the varieties in Europe is shown in Figure 4. We observed that var. *bicolor* tends to be restricted to central-eastern Europe. The varieties a*lbiflorus* and *coccineus* are more widespread, with var. *albiflorus* prevailing in the south and south-east of Europe, and var. *coccineus* in the centre-north of Europe.

**Figure 5:**
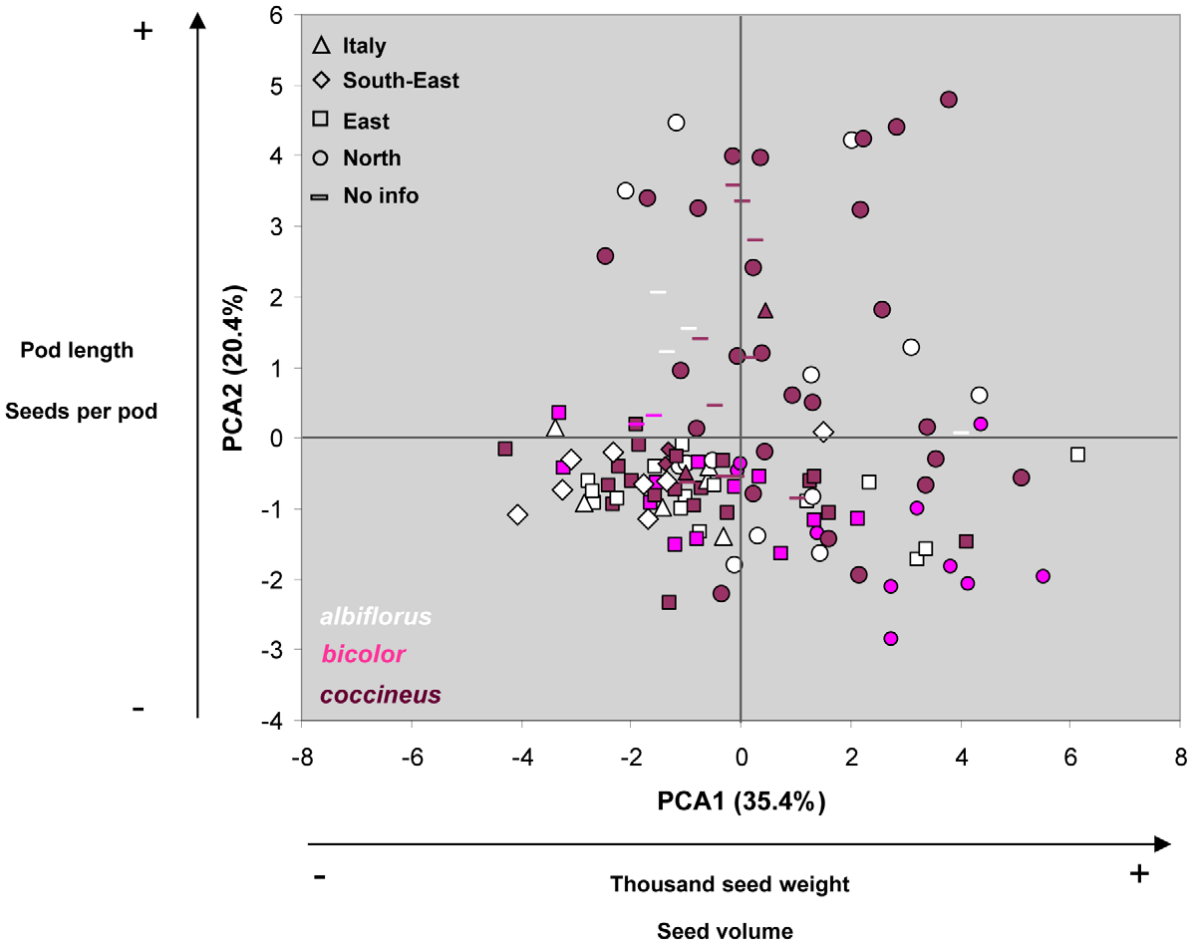
PCA analysis based on 260 accessions and 12 quantitative traits. The percentage of variation explained by each component is given next to the axis. Each accession is marked according to its membership to the different European regions. Varieties are also highlighted according to the colours given in Figure 4.

The PCA performed on 12 quantitative traits showed differentiation of the European accessions from the Italian, southern and south-eastern regions from those of the northern regions (Figure 5). The traits that better explain the PCA1 are seed volume (r  =  0.91, P <0.0001) and thousand-seed weight (r  =  0.82, P <0.0001). The PCA2 mainly positively correlates with pod length (r  =  0.92, P <0.0001) and number of seeds per pod (r  =  0.90, P <0.0001). Accordingly, accessions from the south-eastern regions (Figure 5, bottom left quadrant) mainly show lower pod length, lower numbers of seeds per pod, and lower seed sizes, than accessions from the north.

PCA also confirmed that the geographic distribution of the varieties is different, with var. *albiflorus* and var. *bicolor* showing higher frequencies in the southern and south-eastern parts of Europe (Figure 5).

## Discussion

As far as we are aware, the present study represents the largest analysis of cytoplasmic diversity of a European collection of *P. coccineus* compared to an American set of the same species. We used cpSSRs, which have been shown to be a powerful tool to assess plant genetic diversity, and phenotypic traits, to investigate possible causes for the patterns of the genetic structure of the European runner bean.

### Comparisons of the cpSSR genetic diversity and structure in America and Europe

Comparing only the domesticated accessions, in America, the cpSSR diversity is higher than in Europe, with a loss of diversity (Δ*H*_E_, 0.13) and a loss of allelic richness (ΔR_S_, 0.06). Thus, based on our data, the expansion of *P. coccineus* into Europe was accompanied by a moderate-to-strong cytoplasmic bottleneck. This shows a striking difference from *P. vulgaris*, for which no loss of cytoplasmic diversity has been found [22], [23].

Similar results were obtained by [17] after the analysis of 66 Italian genotypes of *P. vulgaris* and *P. coccineus* by nuSSRs and cpSSRs. They concluded that there is higher nuclear and chloroplast diversity in the common bean compared to the runner bean, which suggested a stronger founder effect for *P. coccineus*.

The bottleneck reported in the present study and the difference in diversity between *P. coccineus* and *P. vulgaris* appear to have arisen because of the introduction of limited genetic diversity into the Old World for *P. coccineus.* Consumers who were more attracted by the various types of seed colour and shape of *P. vulgaris* might have favoured the capture of different alleles and genotypes [11].

The occurrence of a nuclear diversity bottleneck in the European *P. coccineus* landraces was reported by [20] (Δ*H*_E_  =  0.33); compared to what we observe in the present study, here there is a reduction in the cytoplasmic diversity by nearly three-fold (Δ*H*_E_  =  0.13). However, in the American sample of *P coccineus*, the cytoplasmic genetic diversity (*H*  =  0.33) was lower than the nuclear genetic diversity (*H*  =  0.55), as is expected due to the different ploidy levels and inheritance [37]. It can be hypothesised also for the runner bean that the ‘sampling’ of *P. coccineus* accessions during the last 500 years was sufficient to capture a greater part of the (lower) plastidial variation, but not that of the (higher) nuclear variation, as previously suggested for common bean [22], [23].

Moreover, for runner bean, the degree of genetic divergence between the continents is stronger for cpSSRs (*F_ST_*  =  0.37; P <0.0001; present study) than for nuSSRs (*F_ST_*  =  0.19; P <0.0001; [20]). This lower nuclear *F*_ST_ is in line with previous results from different plant species, which have shown how maternally inherited markers (such as cpSSRs) can be in stark contrast to biparentally inherited nuclear markers (such as nuSSRs) when genetic diversity and gene flow are investigated [37]. In particular, the chloroplast has a haploid genome, and it has 1/2N_e_ compared to the diploid nucleus. Thus, for a given intensity of bottleneck, a stronger population subdivision is expected.

However, our data might also suggest that the American sample studied is not fully representative of the American diversity, with a consequent under-estimation of the bottleneck, and a bias (most likely as an over-estimation) in the evaluation of divergence (*F*_ST_) among countries.

Still, and interestingly, a high proportion of the European sample (72.2%) was assigned to the same cytoplasmic group as two accessions from Michoacán (Mexico). The European accessions from groups A1 and A2 (27.8%) appear to have a more composite origin, as these are related to accessions from Costa Rica, Guatemala, Honduras and Mexico. Previous studies indicate Mexico, Guatemala and Honduras as the countries of origin of *P. coccineus* [4]. Angioi *et al.* [7] proposed that two domestication events might have taken place for this species, based on the occurrence of two different wild genetic groups that parallel the differentiation between two groups of domesticated accessions. Based on our data, the existence of (at least) two domestication events is also suggested. Indeed, groups A1 and A2 contain both wild and domesticated accessions of *P. coccineus*. However, a more focused study will be needed to fully resolve this question.

### Level and structure of cpSSR genetic diversity in Europe

Although the genetic diversity was higher in the Iberian Peninsula and Italy (*H_E_*  =  0.31, I_nor_  =  0.61) compared to other regions, similar levels were observed over all of Europe (mean *H_E_*  =  0.28, mean *I_nor_*  =  0.62). Using nuSSRs, [20] observed higher diversity levels (*H_E_*  =  0.36), while an analysis conducted with three nuclear markers and morphological traits on Italian landraces [18] showed genetic diversity estimates similar to our data (*H*  =  0.25). Results obtained with AFLPs on Polish landraces showed lower genetic diversity (*H*  =  0.16; [19]). These differences are likely to be associated to the different types of molecular markers used, as shown by [38]–[40].

The low *F_ST_* values obtained for the cpSSRs among the countries (0.04) and the widespread distribution of the accessions belonging to the five BAPS cpSSR genetic groups, suggest relevant gene flow (seed exchange) of *P. coccineus* in Europe. Indeed, as also observed by [20] using nuSSRs, we observed a significant and positive correlation between cpSSR genetic distances and geographic distances. This might suggest the existence of an isolation-by-distance mode of dispersal.

### Structure of variation for morpho-phenological traits in Europe

Taxonomists have described three botanical varieties of *P. coccineus*, namely var. *albiflorus*, var. *bicolor* and var. *coccineus*, based on the colour of the flowers, which is correlated to the colour of stems and seeds, and to the seed colour pattern [9].

Nonetheless, at the molecular level, we did not observe any significant associations between the botanical varieties and the genetic groups, neither at the cytoplasmic nor at the nuclear level. Moreover, among the 12 quantitative traits investigated, the varieties were different only for pod length and seed volume, which are traits that have no taxonomic value. In agreement with this, other studies have shown no significant associations between flower colour and phenotypic traits, such as number of seeds per pod and seed yield, size and weight [1], [9]. Thus, the botanical varieties do not correspond to three well-defined phylogenetic entities, as previously indicated for morphological intra-species classifications of cultivated plants [10]. Nonetheless, such diagnostic systems remain essential for orientation in the morphological diversity, even though they might be without phylogenetic significance [41].

The European geographic distribution of the varieties showed that var. *bicolor* accessions are prevalently present in central Europe, while, except for Georgia, var. *albiflorus* and var. *coccineus* tend to be present more in southern and northern Europe, respectively. Accordingly, [1] did not find var. *bicolor* on the Iberian Peninsula, where most of the landraces belong to var. *albiflorus* (71%). At the same time, [18] and [41] noted a high prevalence of white-seeded accessions of runner bean in Italy. A possible explanation for this distribution is the higher resistance to low temperatures of the varieties with purple/ violet flowers (var. *coccineus*) than the varieties with white seeds (var. *albiflorus*) [9], [18]. Likewise, consumers from southern Europe appear to prefer the white seeds, given their better culinary quality, higher proportion of total sugars, and lower content of protein and starch [1], [42]. Based on seed size, Acampora *et al.* [18] also suggested the presence of on-farm selection in Italy.

We observed that the accessions characterised by lower seed volume, lower seed weight, shorter pod, and lower number of seeds per pod are mainly disseminated in Italy and in the southern, southeastern and eastern European regions. Additionally, different cpSSR genetic groups show significant phenotypic differences. Indeed, the accessions belonging to group A, which predominate at higher latitudes in Europe, showed shorter pods and lower seed size. No associations were found between nuSSRs and phenotypic traits, as also between the cpSSR and nuSSR genetic groups. Thus, associations between the cpSSR genetic groups and the phenotypic traits might be due to more frequent long-distance pollen flow compared to seed flow, which might occur mostly within short geographic distances. Indeed, according to this hypothesis, the presence of a larger structure at chloroplast level compared to that for nuclear markers would be expected. Our explanation implies that the geographic structure is then associated to adaptive traits that determine the geographic distribution of *P. coccineus* diversity. Thus, association mapping studies aimed at exploiting the diversity of these landraces should carefully take into account the population structure information, and particularly that based on cpSSRs.

In plants with a wide distribution, phenological traits and adaptive molecular variance can be expected to vary clinally along climatic gradients [43], [44]. As examples, latitudinal clines in flowering time have been predicted and shown in accessions of the annual *Arabidopsis thaliana* [43]. Here, we provide evidence of a significant latitudinal cline in the interval of flowering among the northern European and southern European landraces of *P. coccineus* when grown under common environmental conditions during seed multiplication by the same genebank (IPK, Germany).

Specifically, we detected a highly significant negative association between the latitude and the interval of flowering, i.e. the northern populations flower for a shorter time compared to the southern populations. Interestingly, this relationship holds when the effects of population structure for cpSSRs and nuSSRs are factored out. Therefore, this correlation between latitude and phenology is not just a consequence of the geographic distribution of the genetic pools. This suggests that selection (probably for photoperiod sensitivity and/or for low temperature), rather than migration and gene flow, also had a role in shaping the population structure of *P. coccineus* in Europe. Indeed, the most likely explanation for our data is that the growing season is short at high latitudes, as negative correlations were also found between latitude and emergence dates and between latitude and flowering time, although these did not reach significance. This selectively constrains the period available for growth and fruit maturation, and would select for rapid development and early flowering and fruit maturation. Similar results have also been shown in the perennial herb *Lythrum salicaria* [45].

Interestingly, Wallace [46] and White and Laing [47] also observed that in the common bean, the balance between the vegetative and reproductive periods is largely determined by the photoperiod sensitivity/ insensitivity, and that landraces that originate from different geographic regions can have different photoperiod requirements, as well as different tolerances to low temperatures. To the best of our knowledge, similar data are not available to date for *P. coccineus.*

## Conclusions

In summary, we have shown that *P. coccineus* experienced a moderate cytoplasmic bottleneck as a consequence of its introduction into Europe. We have also shown that the European germplasm strongly differentiates from the American reference sample. Founder effects might explain this shift in allelic and haplotype frequencies between the two continents. However, there is evidence that selection might have had a role in the evolution of the European runner bean, as suggested by the geographic patterns of a putative adaptive trait, such as the interval of flowering. Thus, as previously noted [20], it appears possible to consider Europe as a secondary diversification centre for *P. coccineus*. On the other hand, by examining cpSSRs, 12 quantitative traits and nuSSRs, we were not able to distinguish between the three botanical varieties, var. *albiflorus*, var. *bicolor* and var. *coccineus*.

In terms of the breeding of *P. coccineus*, overall, our data suggest that future association mapping studies on European landraces of *P. coccineus* must take into account the population structure for the chloroplast genome. Additionally, the latitudinal gradient for flowering interval indicates that natural selection mapping will be an interesting complement.

## Supporting Information

Figure S1 **UPGMA trees based on the Kullback-Leibler distance, as obtained from the BAPS analysis.** The most probable numbers of populations are indicated for both cpSSRs (**A**) and nuSSRs (**B**). The 21 populations detected by the cpSSR data are assigned to 5 main clusters, while the 24 populations detected by the nuSSR data are assigned to 6 main clusters.
(TIF)

Table S1 **List and details of the 380** ***P. coccineus*** **accessions.**
(XLS)

Table S2 **Mean values of the phenotypic traits used for association analyses.** Data were obtained from an average of 3 years field trials per accession. Missing data are indicated by dots.
(XLS)

Table S3 **Number of alleles and Nei’s gene diversity (HE) per primer pair as detected overall and within countries.** Within brackets: number of private alleles.
(XLS)

Table S4 **Comparison of the genetic structure observed within each continent.** Contingency analyses were performed to compare the chloroplast and nuclear groups for the different Ks.
(XLS)

Table S5 **Results of multiple regression test.** Latitude and genetic structure (both chloroplastic and nuclear) were regressed over flowering interval.
(XLS)
