# Supplementary figures and images for: European Phaseolus coccineus L. landraces: Population Structure and Adaptation, as Revealed by cpSSRs and Phenotypic Analyses
Source: PLoS One. 2013 Feb 22;8(2):e57337. doi: 10.1371/journal.pone.0057337 (PMC3579852; doi:10.1371/journal.pone.0057337)

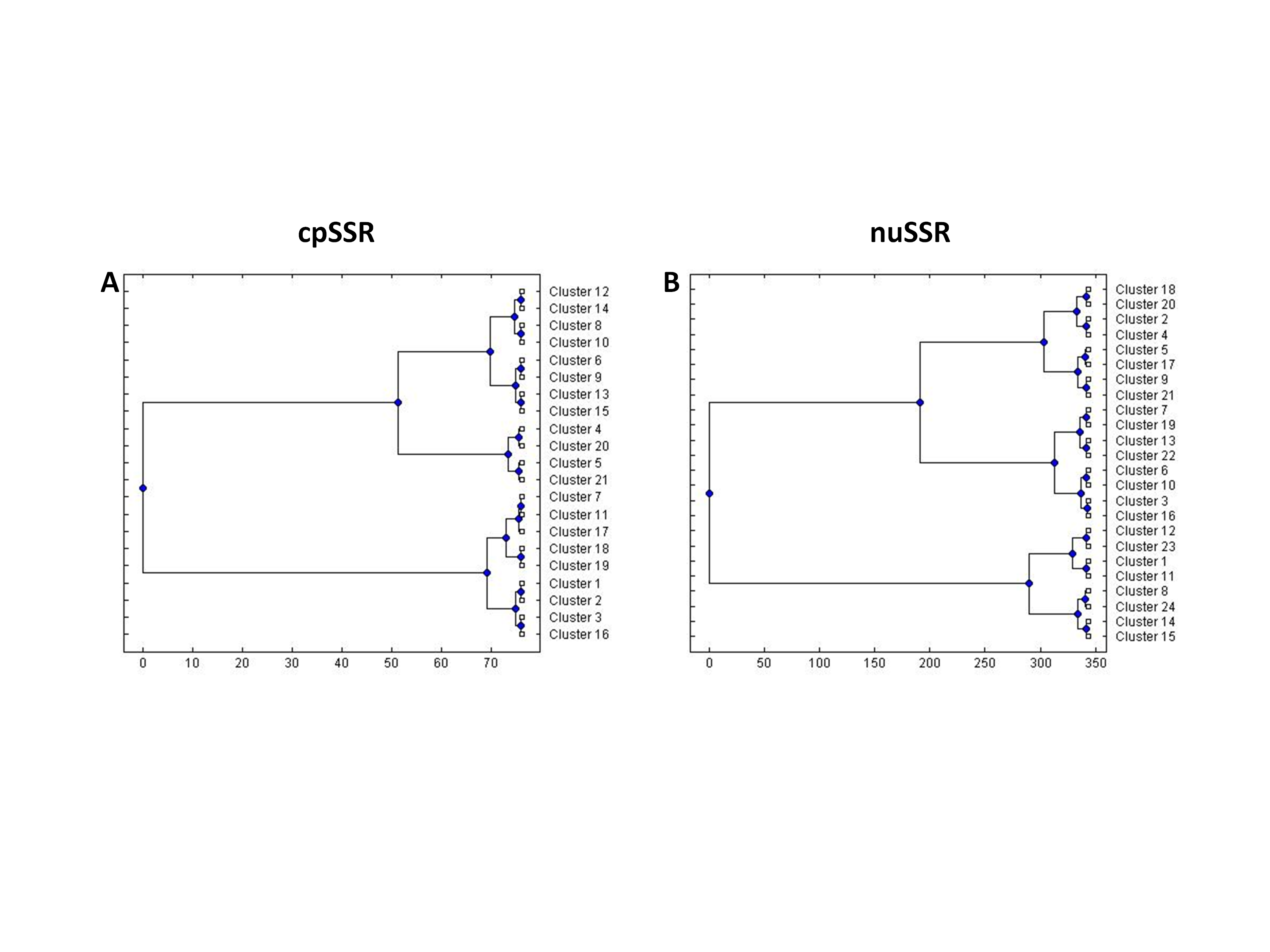

Supplement: Figure S1 — UPGMA trees based on the Kullback-Leibler distance, as obtained from the BAPS analysis. The most probable numbers of populations are indicated for both cpSSRs (A) and nuSSRs (B). The 21 populations detected by the cpSSR data are assigned to 5 main clusters, while the 24 populations detected by the nuSSR data are assigned to 6 main clusters. (TIF) [file pone.0057337.s001.tif]
